# Detecting separate time scales in genetic expression data

**DOI:** 10.1186/1471-2164-11-381

**Published:** 2010-06-16

**Authors:** David A Orlando, Siobhan M Brady, Thomas MA Fink, Philip N Benfey, Sebastian E Ahnert

**Affiliations:** 1 Department of Biology and IGSP Center for Systems Biology, Duke University, Durham, NC, USA; 2 Whitehead Institute for Biomedical Research, 9 Cambridge Center, Cambridge, MA, USA; 3 Department of Plant Biology and Genome Center, University of California, Davis, CA, USA; 4 INSERM U900 and CNRS UMR 144, Institut Curie, Paris, France; 5 London Institute for Mathematical Sciences, London, UK; 6 Theory of Condensed Matter Group, Cavendish Laboratory, University of Cambridge, UK

## Abstract

**Background:**

Biological processes occur on a vast range of time scales, and many of them occur concurrently. As a result, system-wide measurements of gene expression have the potential to capture many of these processes simultaneously. The challenge however, is to separate these processes and time scales in the data. In many cases the number of processes and their time scales is unknown. This issue is particularly relevant to developmental biologists, who are interested in processes such as growth, segmentation and differentiation, which can all take place simultaneously, but on different time scales.

**Results:**

We introduce a flexible and statistically rigorous method for detecting different time scales in time-series gene expression data, by identifying expression patterns that are temporally shifted between replicate datasets. We apply our approach to a *Saccharomyces cerevisiae *cell-cycle dataset and an *Arabidopsis thaliana *root developmental dataset. In both datasets our method successfully detects processes operating on several different time scales. Furthermore we show that many of these time scales can be associated with particular biological functions.

**Conclusions:**

The spatiotemporal modules identified by our method suggest the presence of multiple biological processes, acting at distinct time scales in both the Arabidopsis root and yeast. Using similar large-scale expression datasets, the identification of biological processes acting at multiple time scales in many organisms is now possible.

## Background

Biological processes in living organisms occur on a vast range of time scales, from 10^-9^s (nanoseconds) to 10^9^s (decades), many of them taking place simultaneously. The advent of high-throughput technologies has given biologists the ability to measure system-wide gene expression, potentially capturing many of these processes at once. As a result, one of the major challenges of biological data analysis is the separation of these processes and their time scales. In many cases it is not even known how many processes underlie the measured signal or what their respective time scales are. These questions are particularly relevant to the field of developmental biology. Developmental studies focus on systems such as animal embryos or plant organs in which processes such as growth, segmentation and differentiation can all take place simultaneously, but on different time scales, complicating the interpretation of expression data.

Here we introduce a method for detecting the presence of different time scales in time-series gene expression data. Our approach is based on two assumptions that hold for many data sets of this type. First, that at least two replicate time-series measurements have been produced. Second, that there is at least one time-dependent process for which the time scale is known. This known process allows us to synchronize the replicates, and is most often the time scale on which the data was measured.

**Figure 1:**
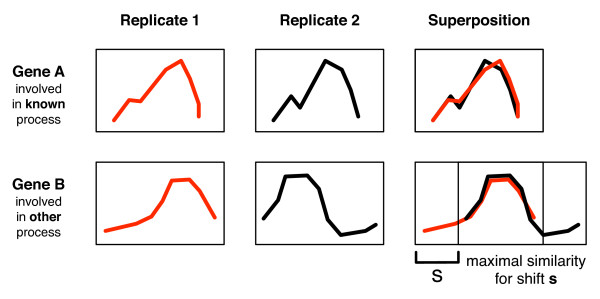
**We search for time scale separation in microarray expression data by detecting patterns that show a temporal shift between experimental replicates relative to the synchronization by a given biological process**. In this example, Gene A is related to the synchronization procedure and shows very similar expression patterns in both replicates. The expression level of Gene B on the other hand is also changing in a reproducible way, but on a different time scale, independent of the synchronization process. By shifting these time series relative to each other and calculating the similarity for each possible shift, we find the superposition that yields the maximal similarity in the overlap window (third column). Because of the varying window size we keep track of the statistical significance (see Methods). The value of the shift *s *which gives rise to the maximal similarity, as well as the statistical significance of this value, allows us to determine whether a given expression pattern is likely to be evidence of a process operating on a separate biological time scale.

If these two conditions are met our method can detect biological processes on time scales other than the known one (that was used to synchronize the replicates) by searching for temporal expression patterns that are similar in the two replicates, but occur at different times. In other words, these patterns are *shifted *(Figure 1). To this end, we measure the correlation of expression patterns adjusted for varying shifts. Assessing the statistical significance of this correlation is not straightforward, as the width of the comparison region varies, depending on the magnitude of the shift. For each gene we can then determine the shift yielding the *most significant *correlation, and rank all genes by this significance to find the most prominent shifted patterns.

As an example of an applicable dataset, consider a gene expression measurement time-series with two replicates that is used to study cell cycle. Both replicates are synchronized in order to start at the same point in the cell cycle. Now let us suppose there is a second time-dependent process that is not affected by the synchronization (ie. not the cell-cycle). The two time-series of cellular snapshots provided by the replicates will now catch this second process in different temporal states. However, to ensure that we are observing the same process in two different states, rather than a signal corrupted by noise, the two snapshots have to be shifted versions of each other with a high degree of similarity, which is why our significance analysis has to incorporate the temporal shift in an explicit manner.

Our approach is somewhat analogous to an astronomical device called the *blink comparator *[1], which is used to distinguish the separate time scales on which planets and stars move across the sky. Two photographs of the night sky are taken on different days and the stars aligned so that they occupy the same position on both photographs. The comparator then alternates between the two images. Any object that is not a star, such as a planet, will jump back and forth, because it moves on a different time scale relative to the stellar background (which only moves due to the Earth's motion). In this analogy, the two astronomical photographs correspond to the biological replicates, the stellar background to the known biological process, and the object which changes position represents another biological process on a different time scale.

We apply our approach to detect time scales in two datasets. The first is a *Saccharomyces cerevisiae *cell-cycle dataset [2], and acts as a benchmark. We demonstrate that our method can successfully detect processes operating on two different time scales, namely real (chronological) time and cell-cycle time. The second dataset measures gene expression through root developmental time in *Arabidopsis thaliana *[3]. Using our approach we discover many classes of statistically significant shifted patterns for the root dataset. These patterns can be further divided into tightly co-expressed spatiotemporal transcriptional modules, some of which are related to processes that occur during branching of the root, termed lateral root initiation. These patterns and modules suggest a rich and complex set of genes that act at multiple time scales to perform a range of biological functions.

## Results

### 1) Detection of separate time scales in *Saccharomyces cerevisiae*

To validate our method we chose to analyze a dataset for which there was a known separation between the time scale of the experiment and the time scale of a biological process of interest. The dataset we chose was a recent synchrony/release time-series microarray dataset from the yeast *Saccharomyces cerevisiae *measuring gene expression through the cell cycle (GEO Accession: GSE8799)[2].

In the synchrony/release protocol used by the study, a population of cells is synchronized to early G1 phase. The cells are subsequently released to progress through the cell cycle, during which a time-series of microarray measurements are made. Thus, the measured time scale in the dataset is chronological time (minutes since release from the synchronization event). However, as the kinetics of release from synchronization always varies from experiment to experiment [4], replicate time-series experiments will not progress through the cell-cycle in exactly the same way. This introduces a separation of time scales between the measured scale, chronological time, and that of a biological process of interest, the cell-cycle.

The dataset itself consists of two replicate synchrony/release time-series experiments, each with 15 Affymetrix Yeast 2.0 microarray measurements taken at 16 minute intervals after the first sample. In this dataset the start of sampling in each replicate began at slightly different times (30 minutes and 38 minutes). As our method requires directly comparable data, simple linear splines were fit to each replicate and sampled at 8 minute intervals starting at 38 minutes after release, with a total of 28 samples per replicate.

**Figure 2:**
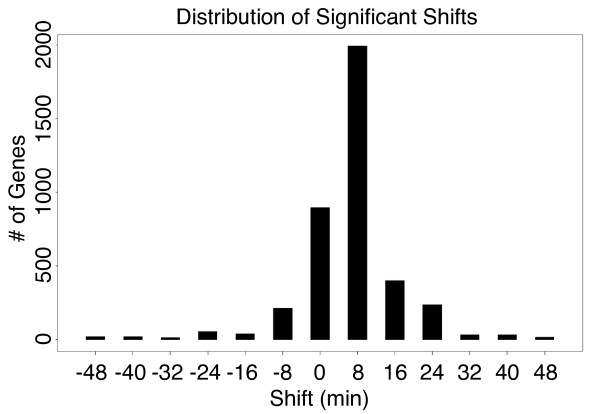
**Distribution of maximally significant shifts**. Our method identified 3974 genes that had significant shifts at or below a p-value of 0.001. Each of these genes was assigned to the shift in which it had the most significant (lowest) p-value.

We analyzed the 5742 probes (each mapped to a gene in [2]) for shifts ranging from -48 to +48 minutes at 8-minute resolution with a p-value at or below 0.001 (Table 1, row 1). We found 3077 genes with a non-zero most significant shift, and ~900 genes that had their most significant shift at zero (Figure 2 and Table 1, row 2).

**Table 1:** Distribution of significant shifts in the cell-cycle dataset

Shift	Not Sig	-48	-40	-32	-24	-16	-8	0	8	16	24	32	40	48
*<*0.001	1768	38	91	182	364	689	1689	2970	3351	2705	1489	441	193	59

Most Sig.	1768	21	19	14	56	41	214	897	1992	402	237	32	33	16

The first row gives the possible types of shifts. The second row gives the number of genes that have a significant shift (p-value < 0.001) at the indicated shift (a gene can have more than one significant shift). The third row gives the number of genes with their maximally significant shift at the indicated shift (a gene can only have one maximally significant shift).

**Figure 3:**
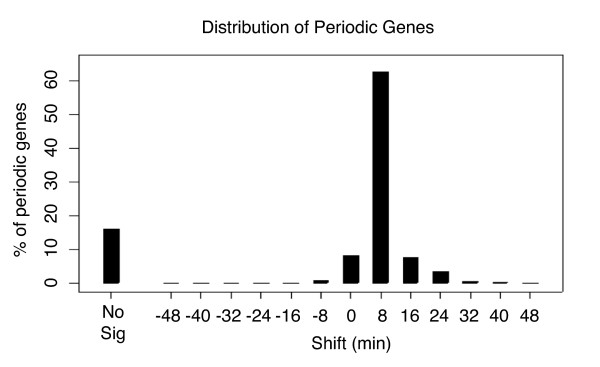
**Distribution of periodic genes identified by Orlando et al. in (Orlando *et al*. 2008)**. Each of the 1274 periodic genes identified by Orlando et al. was assigned to its maximally significant shift. Genes which did not have a significant p-value (< 0.001) were assigned to the "Not Significant" category.

We expected to find a large group of shifted genes related to the cell-cycle given the known time scale separation in this data. We therefore tested if any of the gene sets, defined by maximal shift, were related to the cell-cycle. Of the 1274 cell-cycle regulated probes identified by [2] over 60% had a significant shift at +8 minutes (referred to as Shift+8 genes), with a majority of the remaining periodic genes not having a significant p-value at any shift (Figure 3). We also performed a GO term enrichment analysis on each of the gene sets to determine if we could confirm this annotation [5]. The GO analysis results (Table 2) showed a huge enrichment for terms related to the cell-cycle (mitotic cell cycle: 1.2e^-16^, cell cycle: 5.7e^-16^, cell division: 4e^-13^) in the Shift+8 gene set, with no other gene sets showing any enrichment for cell-cycle related terms. Only two other gene sets, Shift+0 and Shift+16, have any enriched terms. The Shift+0 set is enriched for terms having to do with general biological processes such as growth and RNA processing (ribosome biogenesis: 1.9e^-32^, ncRNA processing: 3.7e^-25^) which are generally not associated with the cell-cycle. This enrichment in the Shift+0 group is not surprising, as upon release from synchrony, cells would be expected to activate programs associated with growth and cellular reorganization. Thus these processes would be operating on the chronological time scale of the synchronization. The GO term analysis revealed that the Shift+16 set was slightly enriched for terms related to catabolic process. The biological relevance of this enrichment remains to be tested, but suggests an additional, as yet, uncharacterized time scale.

**Table 2:** GO term analysis result for cell cycle data

+0 min		+8 min		+16 min	
ribosome biogenesis	1.9 e−32	M phase of mitotic cell cycle	4.3 e−18	proteolysis involved in cellular pro- tein catabolic process	6.9 e−04

ribonucleoprotein complex biogene- sis and assembly	6.5 e−28	mitosis	6.8 e−17	ubiquitin-dependent protein catabolic process	9.7 e−04

ncRNA processing	3.7 e−25	mitotic cell cycle	1.1 e−16	modification-dependent protein catabolic process	9.7 e−04

rRNA processing	1.7 e−20	cell cycle	5.7 e−16		

rRNA metabolic process	2.2 e−19	cell cycle phase	1.1 e−15		

ncRNA metabolic process	2.5 e−17	M phase	2.5 e−14		

maturation of 5.8S rRNA	1.6 e−14	cell cycle process	7.4 e−14		

maturation of 5.8S rRNA from tri- cistronic rRNA transcript	1.6 e−14	cell division	4.0 e−13		

RNA processing	1.3 e−13	DNA replication	2.6 e−09		

ribosomal large subunit biogenesis	6.3 e−13	chromosome segregation	1.5 e−07		

organelle organization	7.1 e−11	sister chromatid segregation	1.5 e−07		

maturation of SSU-rRNA from tri- cistronic rRNA transcript	7.2 e−10	response to DNA damage stimulus	1.6 e−07		

maturation of SSU-rRNA	7.2 e−10	mitotic sister chromatid segregation	1.7 e−07		

endonucleolytic cleavage in ITS1 to separate SSU-rRNA ...	3.9 e−09	glycoprotein metabolic process	2.5 e−07		

cleavages during rRNA processing	5.9 e−09	carbohydrate metabolic process	4.5 e−07		

endonucleolytic cleavages during rRNA processing	8.8 e−09	protein amino acid glycosylation	1.5 e−06		

endonucleolytic cleavage of tri- cistronic rRNA transcript ...	8.8 e−09	biopolymer glycosylation	1.5 e−06		

RNA metabolic process	1.0 e−07	glycosylation	1.5 e−06		

maturation of LSU-rRNA from tri- cistronic rRNA transcript ...	6.6 e−07	glycoprotein biosynthetic process	2.6 e−06		

maturation of LSU-rRNA	6.6 e−07	cellular response to DNA damage stimulus	5.9 e−06		

rRNA 5'-end processing	1.5 e−06	DNA repair	1.3 e−05		

ncRNA 5'-end processing	1.5 e−06	microtubule-based process	2.4 e−05		

RNA 5'-end processing	3.3 e−06	DNA metabolic process	2.8 e−05		

cellular component organization	5.8 e−06	DNA-dependent DNA replication	5.9 e−05		

endonucleolytic cleavage to gener- ate mature 5'-end ...	6.9 e−06	response to stimulus	1.1 e−04		

ribosomal large subunit assembly and maintenance	1.0 e−05	response to stress	3.2 e−04		

ribosome assembly	5.1 e−05	DNA packaging	4.0 e−04		

endonucleolytic cleavage in 5'-ETS of tricistronic ...	6.2 e−05	microtubule cytoskeleton organiza- tion	5.3 e−04		

ribosomal subunit assembly	6.6 e−05				

nucleobase, nucleoside, nucleotide and nucleic acid ...	1.8 e−04				

Every set of genes, defined by the position of their maximally significant shift, was analyzed for over-represented GO terms (*Boyle *et al. 2004). Each shift (column) and term (row) found to be significant are shown.

The identification of biologically coherent sets of shifted genes strongly suggests that our method is able to successfully detect the presence of processes occurring on multiple unrelated time scales. Furthermore, by analyzing the identified genes associated with those shifts, we were able to correctly identify the other major process, associated with Shift+8, as the cell-cycle.

### 2) Detection of separate time scales in the Arabidopsis root

The Arabidopsis root is an excellent model system for studying development due to its simple physical structure. In the root, the majority of new cells are born at the root apex from a stem cell population that surrounds the quiescent center (QC). Cell types are constrained within files, and with each new cell division, an older cell is successively displaced distal to the stem cell population. A cell's developmental time line can therefore be tracked along the root's longitudinal axis. In the work of Brady et al. [3], two developmental microarray time courses were generated by taking 12 or 13 successive transverse sections along an Arabidopsis root (GEO Accession: GSE8934). We use these two time-courses as the replicates (removing the 1^st ^section of the 13 section time-course) and use developmental time as the known time scale.

**Figure 4:**
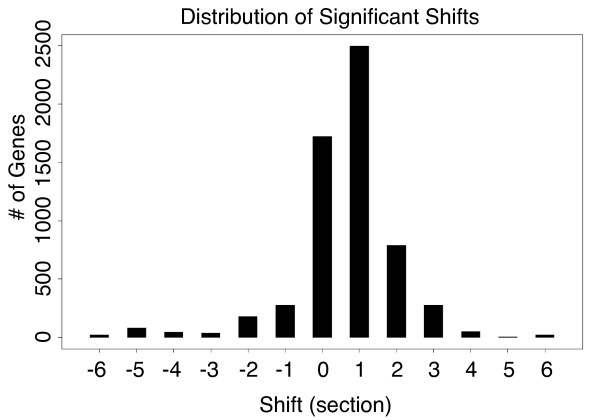
**Distribution of maximally significant shifts in the root dataset**. Our method identified 5592 genes that had significant shifts at or below a p-value of 0.01. Each of these genes was assigned to the shift in which it had the most significant (lowest) p-value.

We analyzed each of the 22746 genes in the root dataset for shifts of -6 to +6 sections. We used a maximal shift threshold of ± 6, as larger shifts would result in an overlap window covering less than half the developmental time points. The distribution of maximally significant shifts for the 5992 genes with shifts at or below a p-value of 0.01 is shown in Figure 4. As sections sampled in both roots were not exact replicates, we find that the mean value of maximal shifts over all genes is 0.63 representing the approximate temporal difference in sections between both root replicates. Therefore, genes with profiles shifted greater than +2 sections and less than -2 were considered for further analysis.

**Figure 5:**
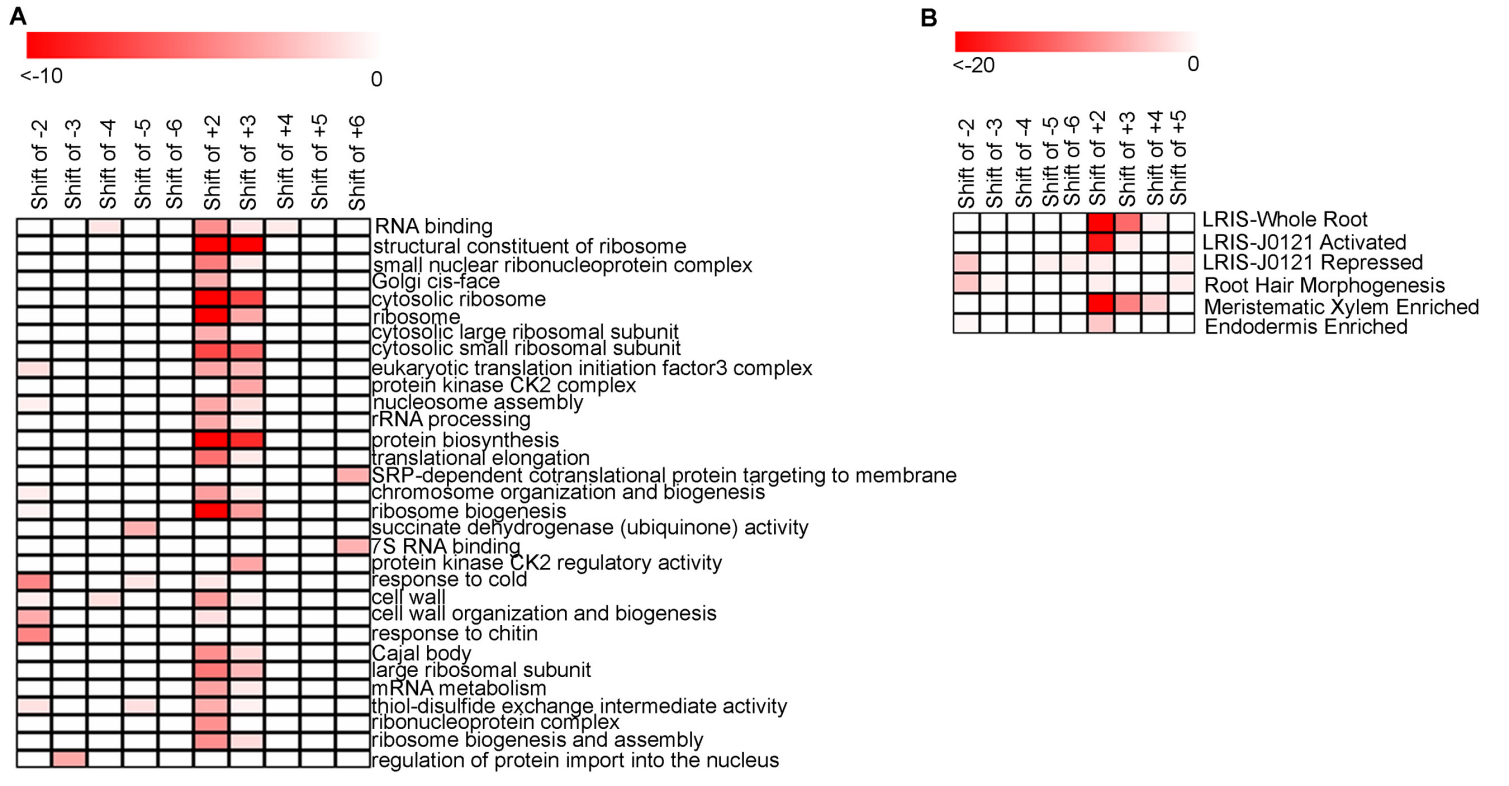
**Gene ontology (GO) terms associated with shifted profiles**. Log_10 _p-value is indicated. (A) All patterns except for shifts of +5 and -6 show enrichment of an associated biological process. (B) Cell-type enrichment, and enrichment of genes associated with auxin-activated lateral root initiation in the lateral root inducible system (LRIS). Genes activated or repressed within xylem pole pericycle cells (J0121) using the LRIS were also enriched.

While genes that display shifted expression profiles in vertebrates are known to regulate processes such as somitogenesis [6] and, as shown above, to regulate the cell cycle in yeast, the biological function of genes exhibiting shifted profiles in the Arabidopsis root was unclear. To infer their function, we tested for statistically significant enrichment of Gene Ontology (GO) terms, and for genes annotated as being associated with biological processes in microarray analyses using the ChipEnrich program [3,7]. These include genes associated with primary or secondary cell wall biosynthesis, with the M-phase or S-phase of mitosis, genes differentially expressed during auxin-activated lateral root initiation (Lateral Root Induction System or LRIS) that are dependent upon auxin signaling, genes differentially expressed in pericycle cells that are competent to differentiate into lateral roots using the LRIS, genes expressed during root hair morphogenesis, and for genes whose expression is enriched in individual root cell types [3,8-13]. This analysis demonstrated that all shifted gene sets, except for the gene sets associated with shifts of +5 and -6, show enrichment of terms associated with at least one biological process (Figure 5A), and some shifted gene sets showed very strong enrichment in an individual cell type (Figure 5B). Also, gene sets with shifts of +2 and +3 sections show very similar term enrichment suggesting either a conservation of biological function between these two groups of genes, or technical noise. It may be the case that the majority of these genes have a true shift of +2.5 sections, which could not be detected based on our method given the resolution in sampling sections.

**Figure 6:**
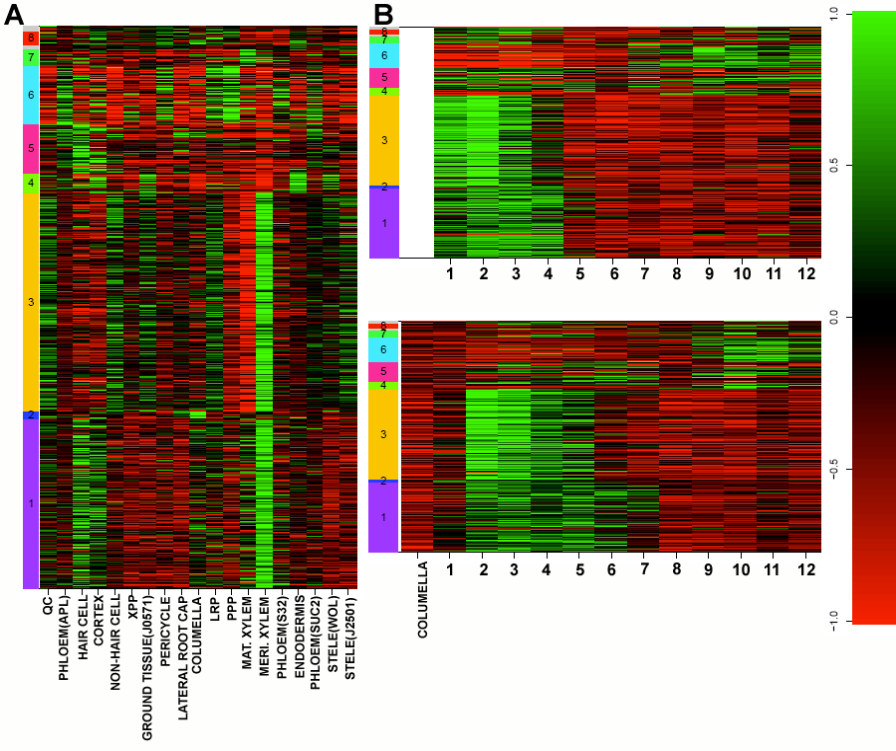
**Clustering of shift profiles identifies spatiotemporally regulated modules of genes for shifts of +2**. (A) Relative expression by marker line is visualized. Clusters of spatially co-expressed genes are indicated on the y-axis. (B) Relative expression by longitudinal section in the two roots is visualized. Root sections are indicated on the x-axis, and clusters from A are further visualized on the y-axis.

**Figure 7:**
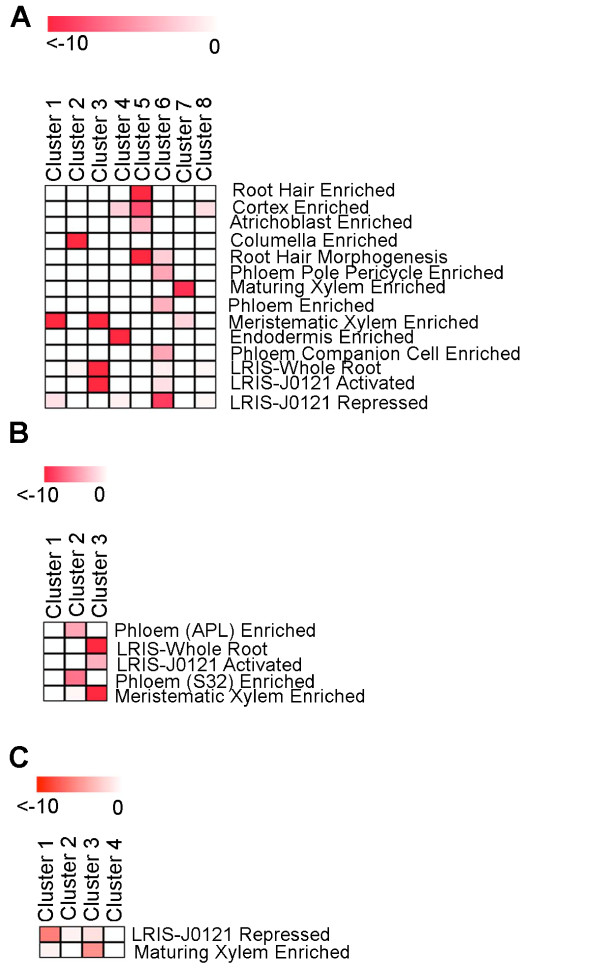
**For cases where multiple spatiotemporally regulatory modules were identified, statistically significantly enriched expression within cell types was also tested**. The p-value scale obtained using the hypergeometric distribution is indicated on the top of each panel. (A) Shift of +2. Many clusters display distinct cell type enrichment profiles. (B) Shift of +3. At least two significantly cell type-enriched clusters were identified. (C) Shift of -2. Among many clusters, specific cell-type enrichment was identified in only a single cluster.

The observation that genes associated with a shifted profile are enriched in single cell types suggests that spatially regulated transcriptional modules may exist. We next attempted to determine if genes associated with a particular shifted profile also showed strong temporal regulation in addition to spatial regulation. First, to systematically separate out these cell type specific modules within each shifted gene set we hierarchically clustered the individual genes on their cell type expression [3], and cut these trees at a Pearson correlation threshold of 0.75. These clusters displayed distinct cell type and developmental stage enrichment (Figure 6-7 and Additional File 1). Resulting clusters with greater than 10 members were queried for GO term and biological process enrichment as described above.

Many different enriched processes were identified in these spatiotemporally co-expressed groups suggesting that they possess distinct biological functions. One cluster of phloem-enriched, chloroplast genome-encoded genes whose expression is shifted -2 sections (Cluster 1, Additional File 1) is associated with the translation of energy capturing proteins. Two clusters that show a shift of +2 also show enrichment of genes known to be activated or repressed during lateral root initiation in the LRIS. Lateral roots develop, at regular intervals, from pericycle cells located at root xylem poles in the root maturation zone, and their initiation is dependent upon xylem pole architecture [14]. Cluster 3 shows an extremely strong enrichment for genes activated during lateral root initiation in the LRIS in both the whole root and activated in pericycle cells located at the xylem pole in the LRIS (p = 2.4e^-113^, p = 3.1e^-39^). These genes also show enriched expression in xylem cells in the meristematic zone (p = 3.2e^-63^) and display a time shift within the meristematic zone (Figure 7A). Interestingly, a second cluster (cluster 6) is enriched for genes repressed during lateral root initiation in xylem pole pericycle cells in the LRIS (p = 1.92e^-9^), but also contains genes whose expression is enriched in phloem cells, phloem companion cells and phloem pole pericycle cells (p = 1.59e^-4^, p = 5.6e^-5^, p = 5.54e^-5^) (Figure 7A). This profile shows a shift in the root maturation zone.

## Discussion

Biological processes on multiple time scales occur during the development and morphogenesis of embryos, tissues and organs. Using time series microarray expression data in replicate, we have developed a method that identifies a number of temporal scales in addition to the time scale being measured. This method was able to identify these time scales in two different organisms, suggesting that it is an organism-independent method.

Given the number of genes in high-throughput datasets, the computational efficiency of any data analysis method is critically important. By converting the data to rank permutations (see Methods), we can use uniformly distributed random permutations as a null model. As a result, our method is able to use a continuous Gaussian distribution for *p*(*γ_i_*) as a close approximation to the real (discrete) probability density function of *γ_i _*values. Using a Monte Carlo simulation over uniformly random permutations we confirmed that this continuous distribution is an accurate approximation (data not shown). Note that, since the Gaussian distribution extends below *γ_i _*= 0, the p-value given by the Gaussian distribution is in fact an upper bound on the true p-value for small (i.e. the most significant) *γ_i_*, which means that the true p-value lies even lower. Therefore, our method provides an efficient, accurate, and conservative method for determining the significance of shifts in high-throughput datasets. Previous work on time-shifted expression data [15-18] has focused on other biological questions, such as the detection of pair wise interactions between genes, rather than on the detection of processes on separate time scales and the comparison of replicates. As a result these approaches do not place an emphasis on shift classification, as they do not explicitly incorporate the shift into their significance analysis [15-17] and only consider small shifts [18]. The shift significance analysis however is crucial to our approach, which aims to detect similar but shifted patterns (see Background section above), and must therefore carefully weigh the relative significance of similarities across varying sizes of the comparison window. Because our approach focuses on comparing replicates, we seek pairs of series that are highly shape-similar across the widest possible comparison window.

In our analysis of the yeast cell-cycle dataset, it is not a coincidence that the cell cycle process was identified in the Shift+8 group and that the original study adjusted the sampling times by eight minutes in the second biological replicate. In the original study, the authors employed a model designed to use auxiliary budding index data to specifically analyze kinetics of populations released from synchrony/release experiments [4]. They used this information to determine which of the samples to hybridize to microarrays. Our method, blind to this prior knowledge, successfully identified this difference as the +8 minute shift. To ensure this agreement was not due to the 8-minute interval data splining used, we repeated the analysis on data splined at one minute intervals (data not shown). This still identified shifts of +8 and +9 minutes as being highly enriched for known periodic probes, thus indicating the method is not sensitive to sampling intervals and successfully detects the known biological shift.

Numerous biological processes have been identified in plants that occur in multiple time scales ranging from seconds (calcium signaling) to hours (circadian rhythms). In the root however, the full spectrum of biological processes that act in multiple time scales has likely not been described, due to a lack of knowledge of the time scales that these processes are acting on. Our rigorous method is able to utilize the gene expression dataset measuring expression through root developmental time in *Arabidopsis thaliana *to identify numerous spatiotemporal transcriptional modules acting in separate time scales. Each spatiotemporal module demonstrates a strong conservation of biological association occurring during root development. Interestingly, the strongest observed associations are linked to genes expressed during the process of lateral root initiation.

Lateral root initiation occurs at regular intervals within pericycle cells located at the xylem pole, suggesting cell-cell communication between xylem and pericycle cells [14]. However, specific causal factors within the xylem have not yet been identified. Furthermore, periodic fluctuations in auxin response activity in xylem cells within the root basal meristem that regulate lateral root initiation in the root's maturation zone have been reported using a synthetic auxin reporter, but no candidate genes have been identified to play a functional role in this process [19]. We propose that genes contained within the two clusters showing a shifted profile of +2 and a corresponding statistically significant enrichment of genes activated or repressed during lateral root initiation in the LRIS may contain some of the previously unidentified factors that play an important role in regulating lateral root initiation. Genes in cluster 3 whose expression is enriched in xylem cells in the meristematic zone may act in the fluctuating auxin response mechanism associated with lateral root initiation, perhaps by signaling to associated pericycle cells. Genes in cluster 6 that are repressed in the xylem pole pericycle upon auxin-induced lateral root initiation are also highly expressed in phloem tissue and phloem pole pericycle cells. No functional link has been established between this tissue and lateral root initiation, but our data suggest that during lateral root initiation, genes that are actively repressed in xylem pole pericycle cells must also be repressed within phloem tissue and within phloem pole pericycle cells.

Our analysis uses p-values in two separate places, which should not be confused. Firstly, they are employed in form of the significance thresholds of p < 0.01 (for *Arabidopsis*) and p < 0.001 (for yeast), which are thresholds for a given shift class, and ensure that each such class contains only a small proportion of false positives. These thresholds are picked for technical reasons, and are therefore inevitably somewhat arbitrary. The second role of p-values is in the subsequent GO enrichment analysis for each class, where they measure the biological significance of the classification. The extremely small p-values we find in this context demonstrate that the shift classification is indeed biologically meaningful.

In principle this method can be generalized to the case of three or more replicates, by choosing *m-1 *independent pairs among the *m *replicates, calculating the relative shifts and their respective p-values for these pairs, and combining the p-values using Fisher's method [20] to find the most significant combination of shifts. The genes can then be classified according to their position in this (*m-1*)-dimensional space. Note that the rapid growth of the volume of this space with *m *is likely to limit the feasibility of this generalization for *m *larger than three or four.

## Conclusions

For all identified, uncharacterized modules in both yeast and Arabidopsis, further studies are needed to determine the relevant time scale of the observed shifts, and the nature of these shifts. Do these shifts act as part of signaling pathways that are on the scale of seconds, as part of metabolic rhythms [21], or are they shifting with respect to the circadian clock? Furthermore, are these groups of co-expressed genes oscillatory in nature, or is their observed shift part of a moving wave of expression that is not oscillatory? How are these temporal shifts generated? Finally, the functional roles of these genes acting at separate scales need to be experimentally elucidated. Regardless, the numerous spatiotemporal modules identified by our method suggest the presence of multiple biological processes, acting at distinct time scales in both the Arabidopsis root and yeast. Using similar large-scale expression datasets, the identification of biological processes acting at multiple time scales in many organisms is now possible.

## Methods

### Significant Shift Detection in Replicates

Consider a pair of replicate datasets with *M *probesets or genes and *N *datapoints, which we write as *M *× *N *matrices d^1^_ij _and d^2^_ij_. We convert these series to rank permutations for each probeset, resulting in two new matrices π^1^_ij _and π^2^_ij_.

As an example with *N *= 4, consider a row in d^1 ^reading '0.3, 0.5, 0.6, 0.2', for which the corresponding rank permutation in π^1 ^would be '3, 2, 1, 4', since 0.6 is the highest value, 0.5 the second highest, and so on. Thus each row of π^1 ^and π^2 ^contains a permutation of length *N*. In the real data ties are highly unlikely due to the high resolution of the measurements, and can be broken randomly if necessary.

The conversion to permutations simplifies the null model considerably, which makes it straightforward to measure the significance of the similarity between the replicates in a computationally efficient way. Rank permutations also form the basis of other correlation measures, such as Spearman's rank correlation [22]. Another advantage of rank-based measures is a lower sensitivity to outliers and a greater emphasis on shape similarity [18].

We then calculate a measure of similarity of two rank profiles for a given shift *s*:

For *s *≥ 0:

For *s *< 0:

For uniformly random permutations (which will occur for any i.i.d. randomly distributed data) we expect γ_i _to follow a Gaussian distribution with mean μ and standard deviation σ, given by:

These expressions follow from the mean and standard deviation of the difference between two uniformly distributed continuous random variables. We have confirmed the accuracy of these distributions using computer simulations (see Discussion). The p-value (confidence value) for a given γ_i _is given by the probability that the same value of γ_i_, or an even rarer value, occurs by chance. Hence the p-value based on the Gaussian distribution, for γ_i _< μ is given by:

We are only interested in similarity, i.e. in cases where γ_i _< μ. Hence we can calculate a p-value for every gene *i *and every shift *s *under this condition. We can then record, for each gene, the shift at which the maximally significant (smallest) p-value occurs, and the p-value itself. Note that the most significant shift between two replicates does not necessarily correspond to the shift with the lowest value of γ_i _itself.

### Biological Process Enrichment - *Arabidopsis thaliana*

All probesets that display a shift with a significance value of p < 0.01 were selected for further analysis. AGI identifiers were assigned to probesets using data from the 2008-5-29 Affy_ATH1_array_elements file. The ChipEnrich program was modified by including genes enriched in individual root cell types as identified by [3]. Genes activated or repressed in xylem pole pericycle cells during lateral root initiation in the auxin-activated Lateral Root Induction System (LRIS) were also included. These were obtained from [12], with activated genes from clusters 1, 2, 3, 5, 9 and 10, and repressed genes from clusters 4, 6, 7 and 8.

## Authors' contributions

DAO performed most of the data analysis and participated in the conception and design of this study, as well as the writing of the manuscript. SMB primarily contributed to the conception and design of this study, and participated in the data analysis and writing of the manuscript. TMAF participated in the study's coordination and design. PNB contributed to the coordination and conception of this project. SEA was primarily involved in the design of this study and the writing of the manuscript. All authors read and approved the final manuscript.

## Supplementary Material

Additional File 1 **Clustering of shift profiles identifies spatiotemporally regulated modules of genes**. A figure showing clustering of shift profiles identifies spatiotemporally regulated modules of genes for shifts of +2, +3, +4, -2 and -5. For all shifts, relative expression by marker line is visualized in the left heatmap, and relative expression by longitudinal section in the two roots is visualized in the right heatmaps. The relative expression scale is visualized on the right. If clusters with greater than ten members were identified, these are indicated on the left side of each heatmap. Click here for file
