# Supplementary figures and images for: Detecting separate time scales in genetic expression data
Source: BMC Genomics. 2010 Jun 16;11:381. doi: 10.1186/1471-2164-11-381 (PMC3017766; doi:10.1186/1471-2164-11-381)

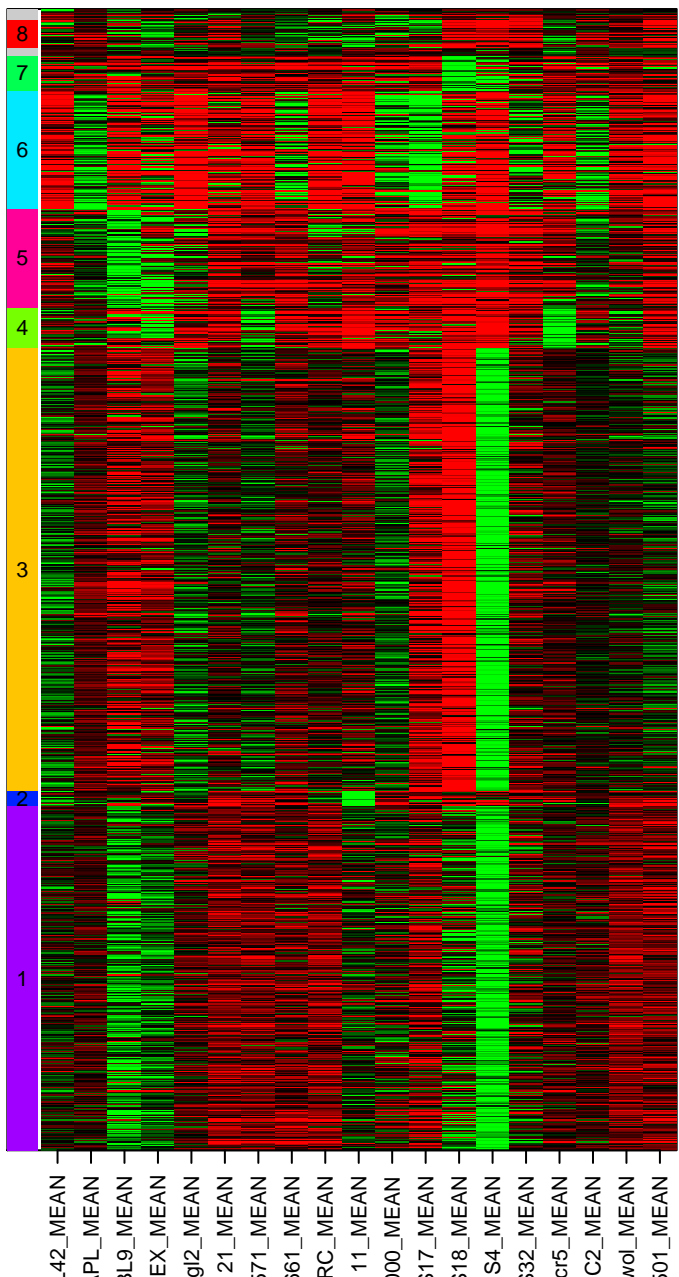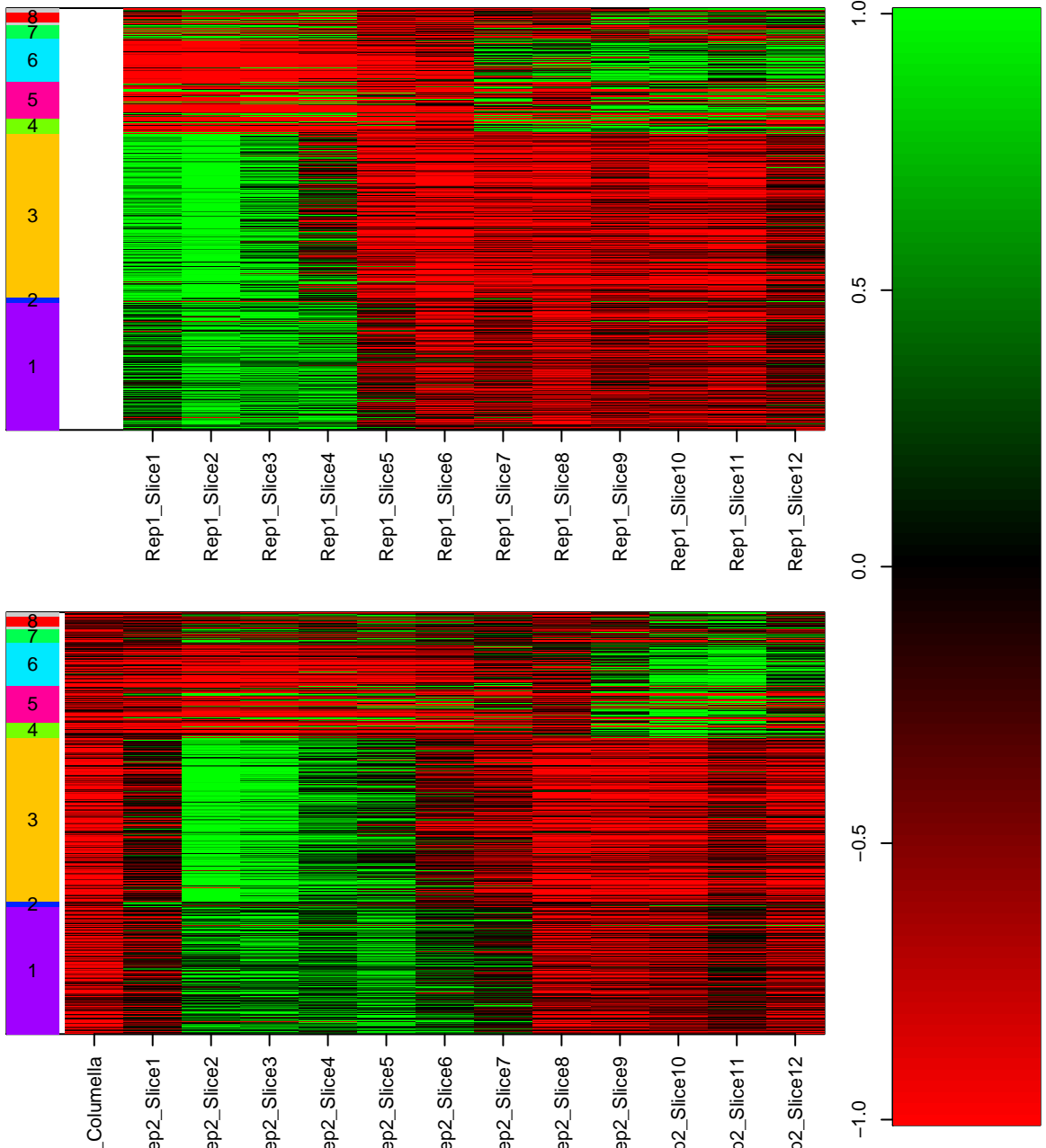

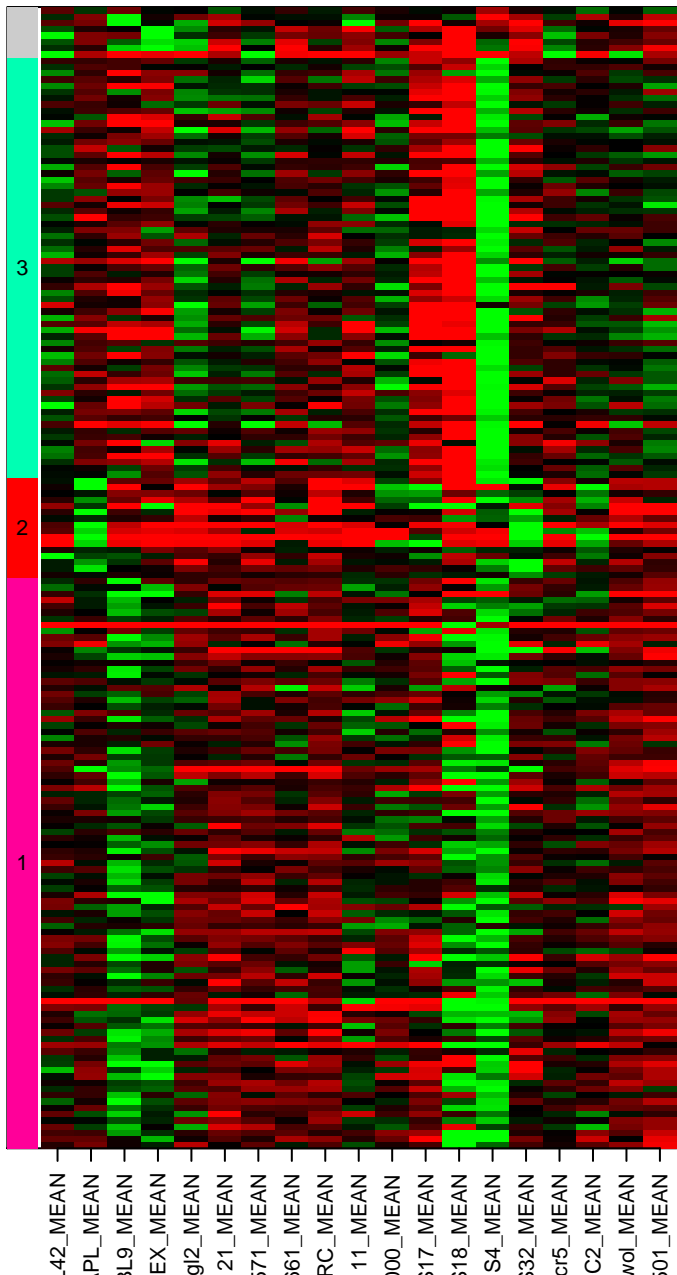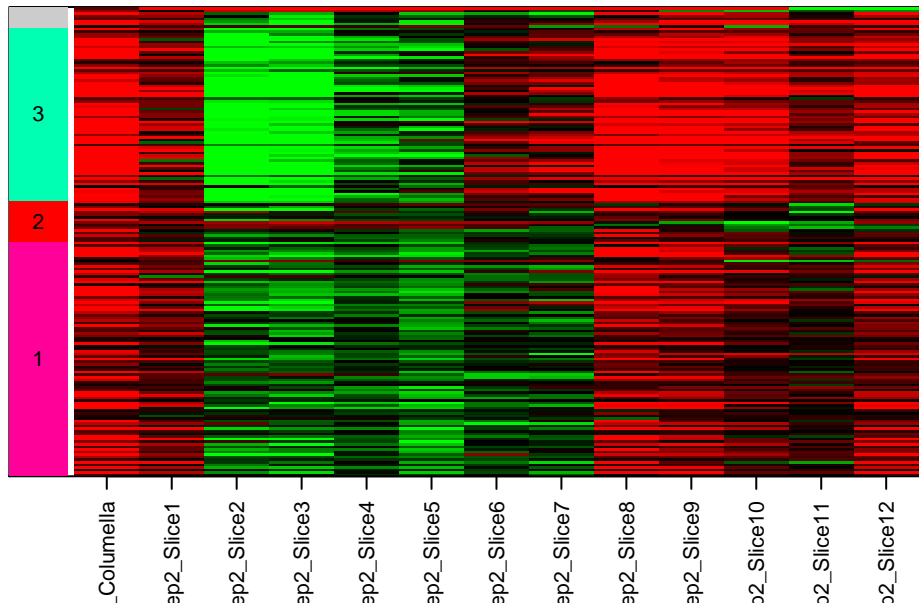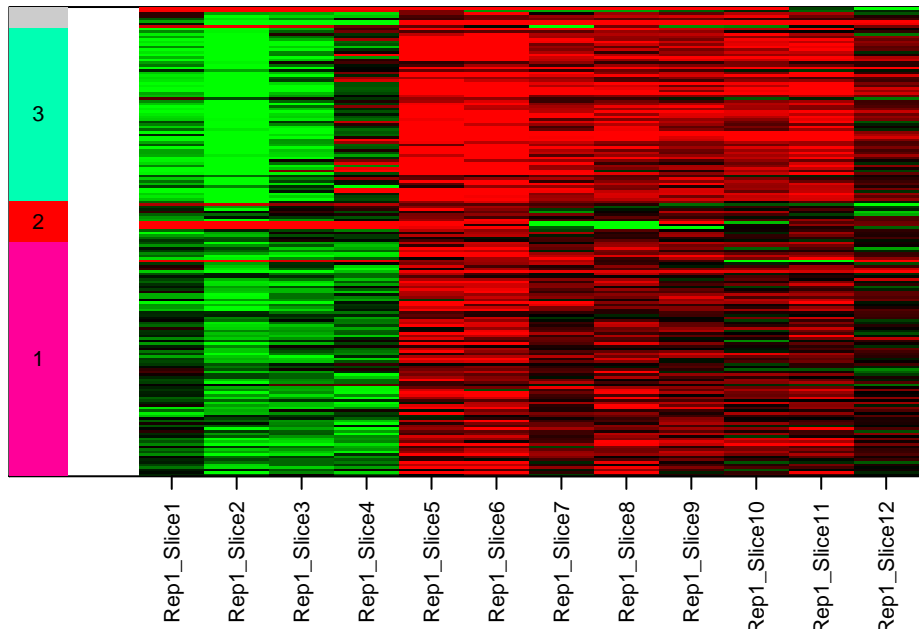

Shift 3

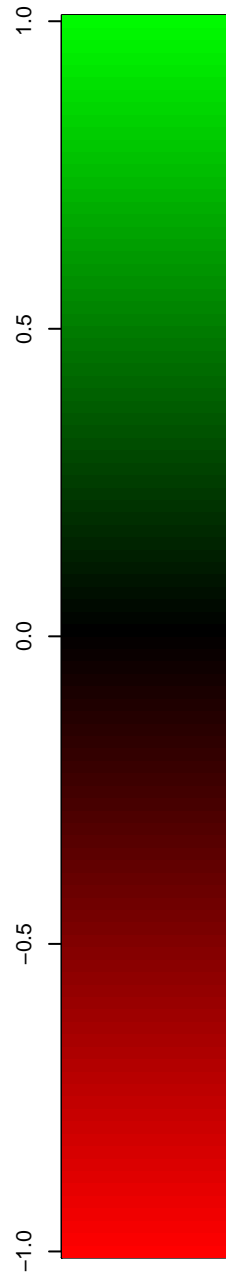

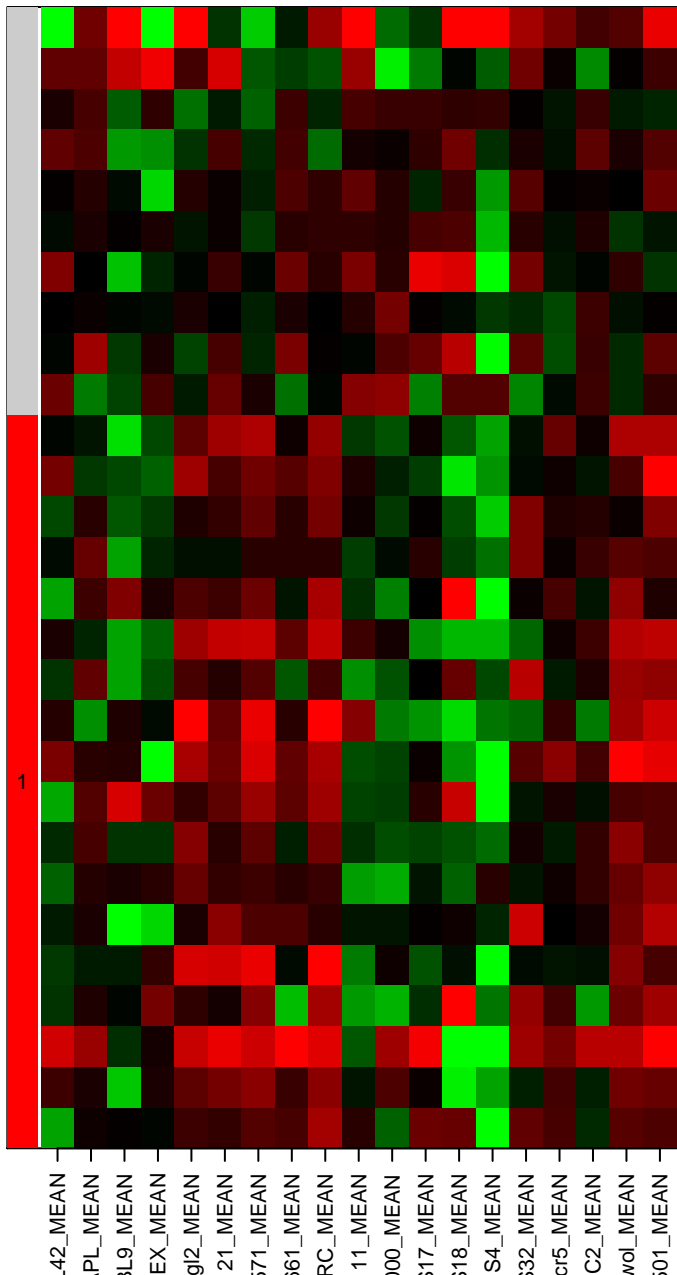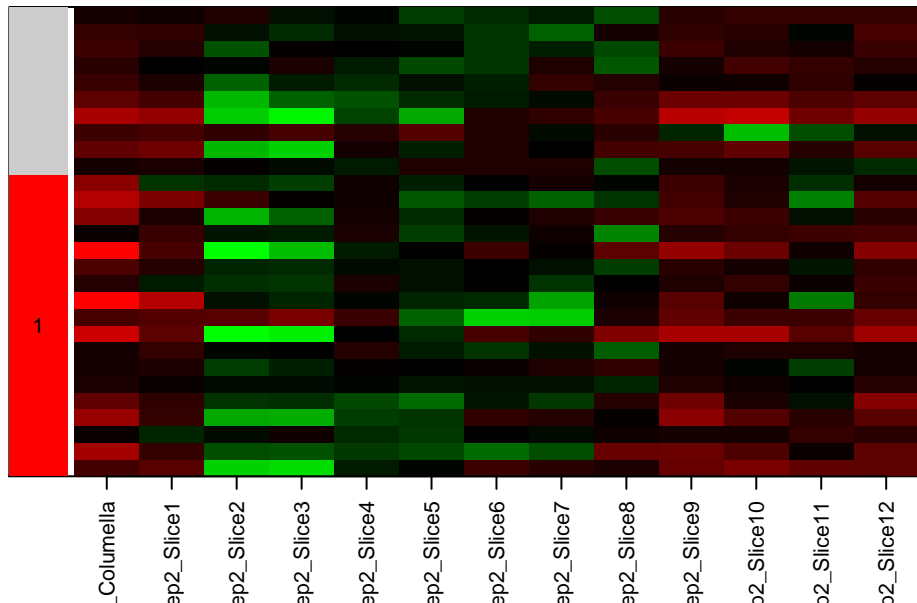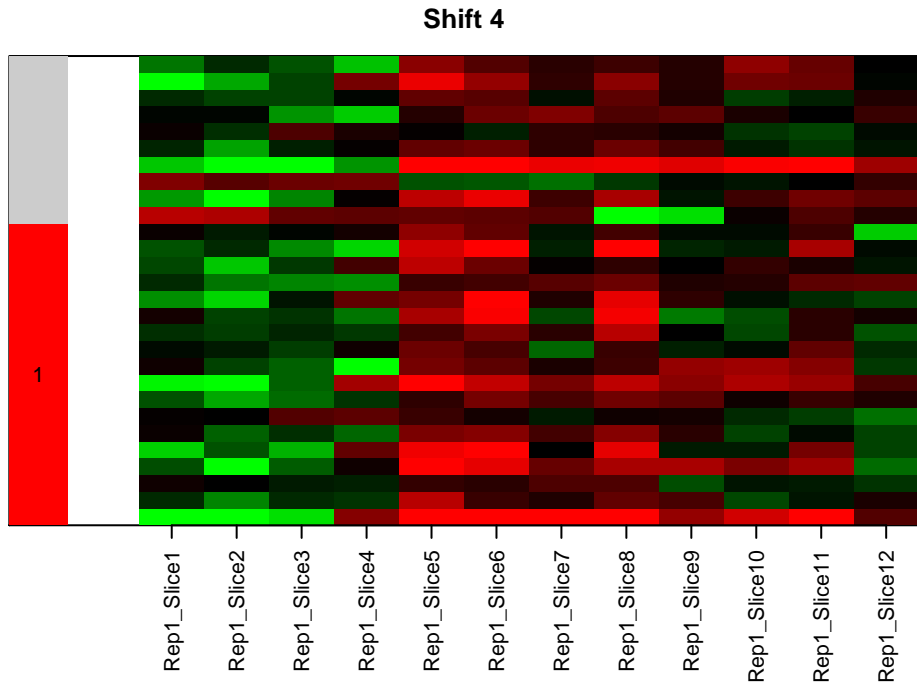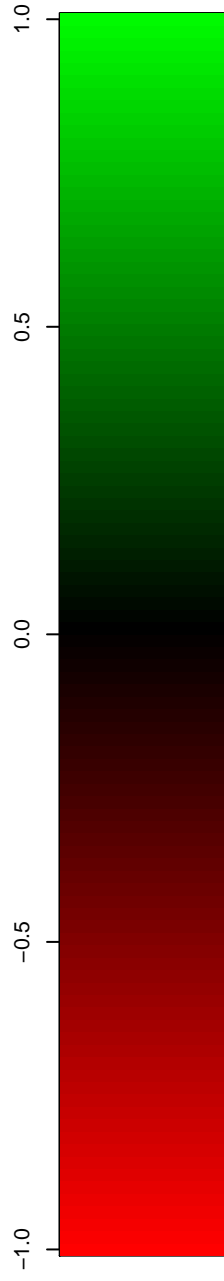

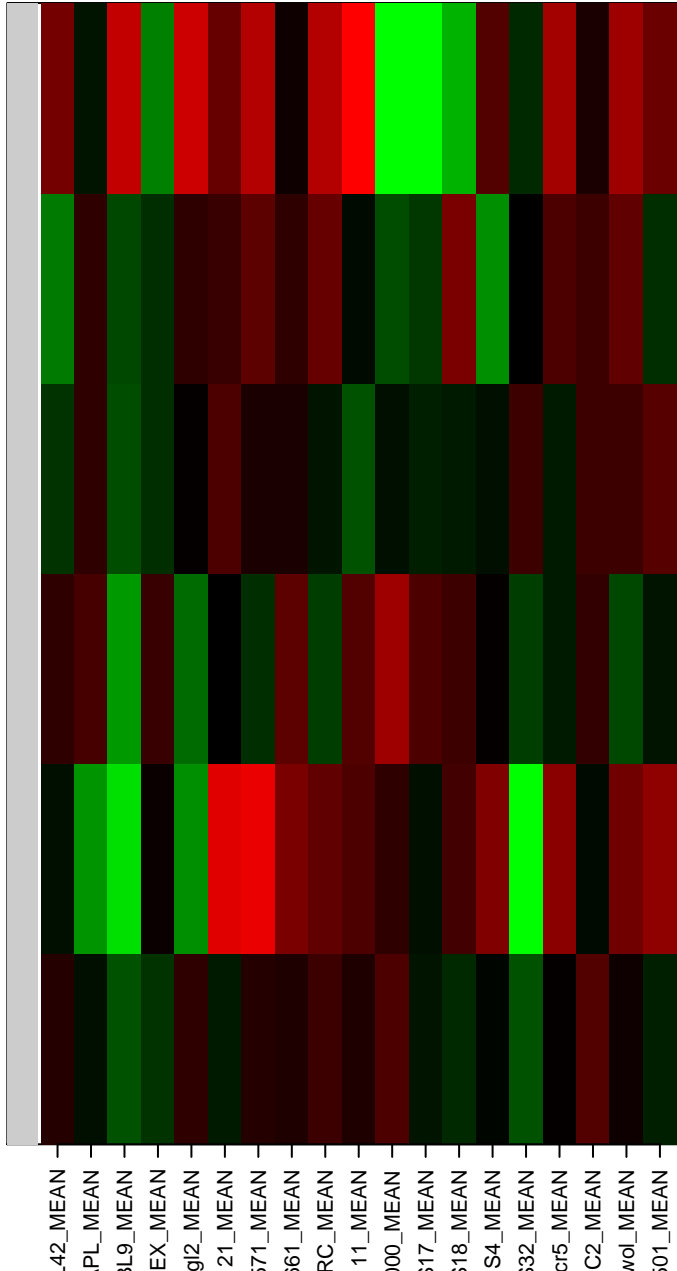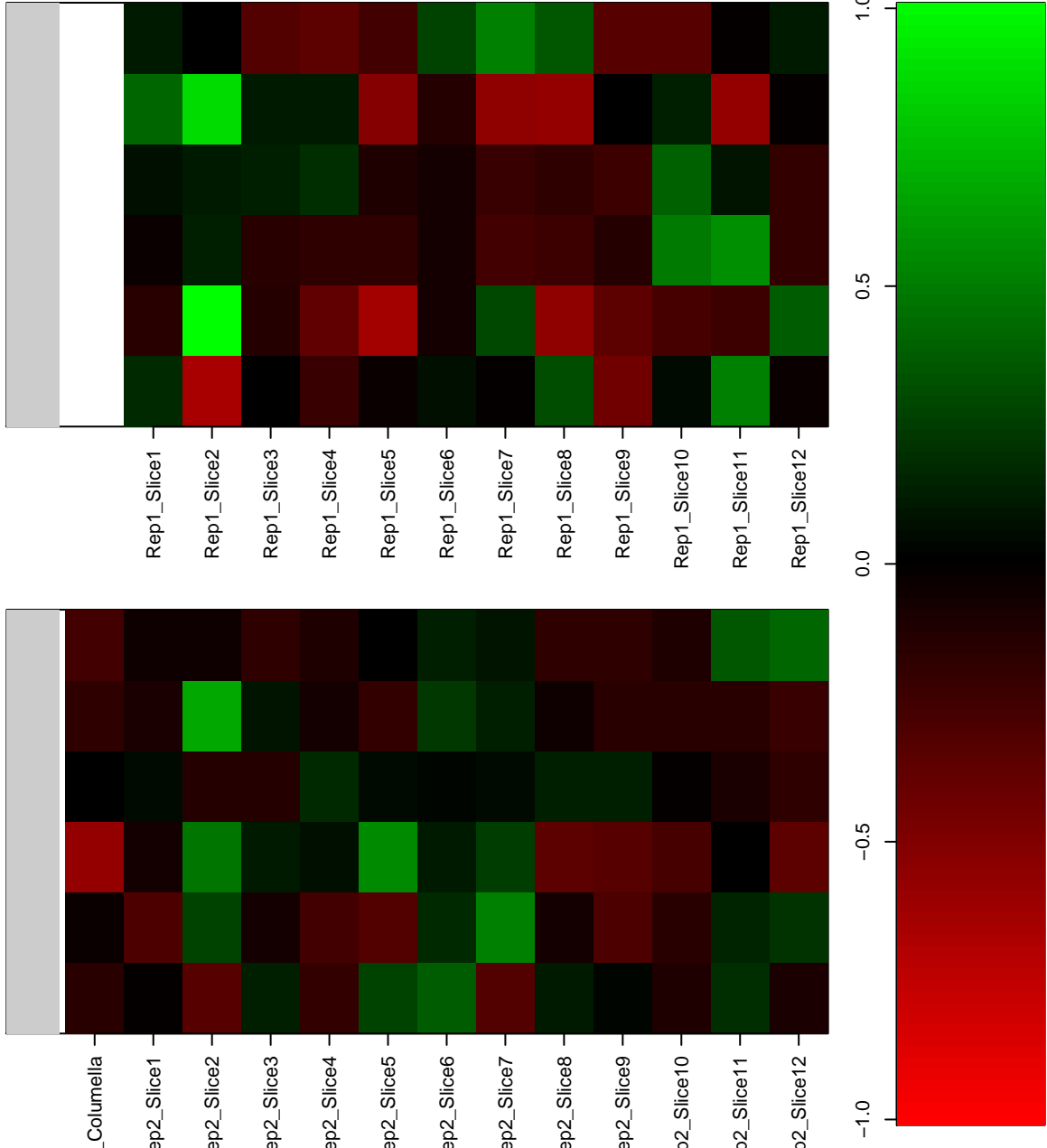

Shift 5

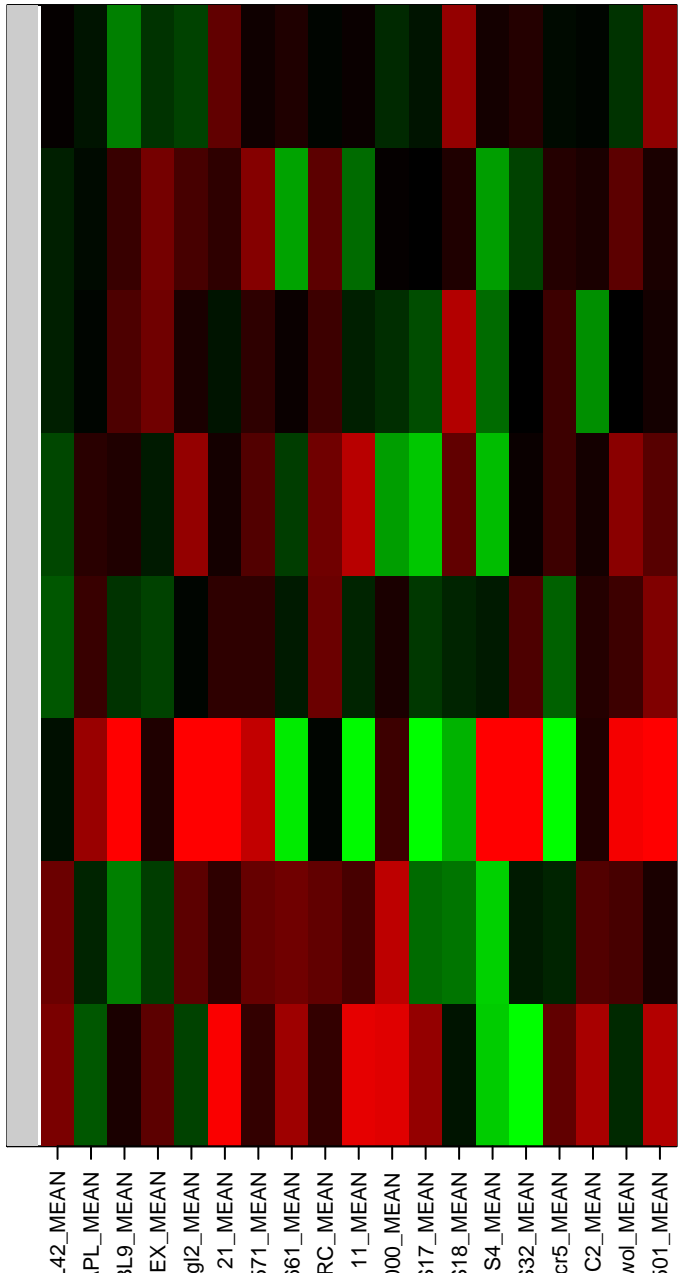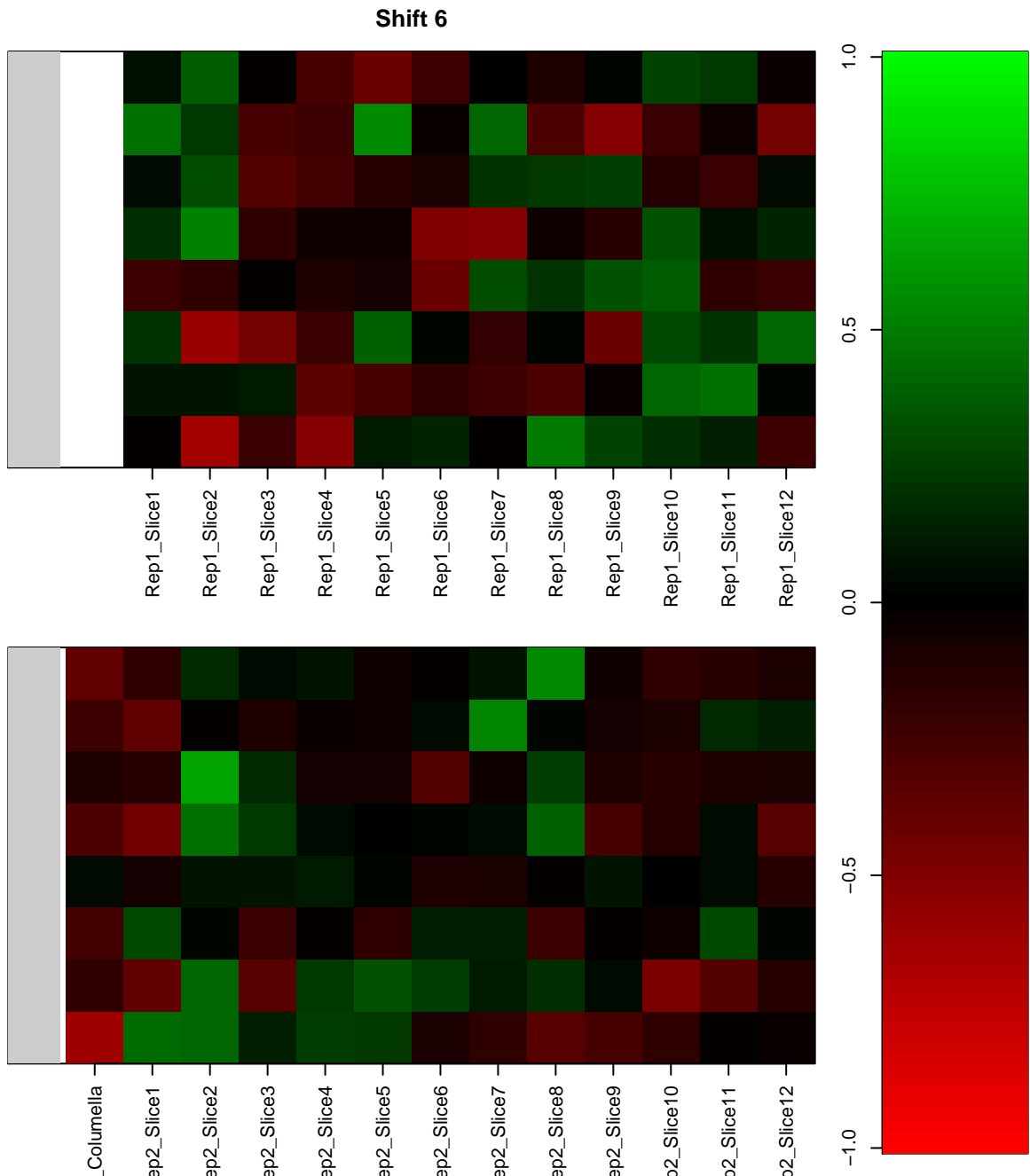

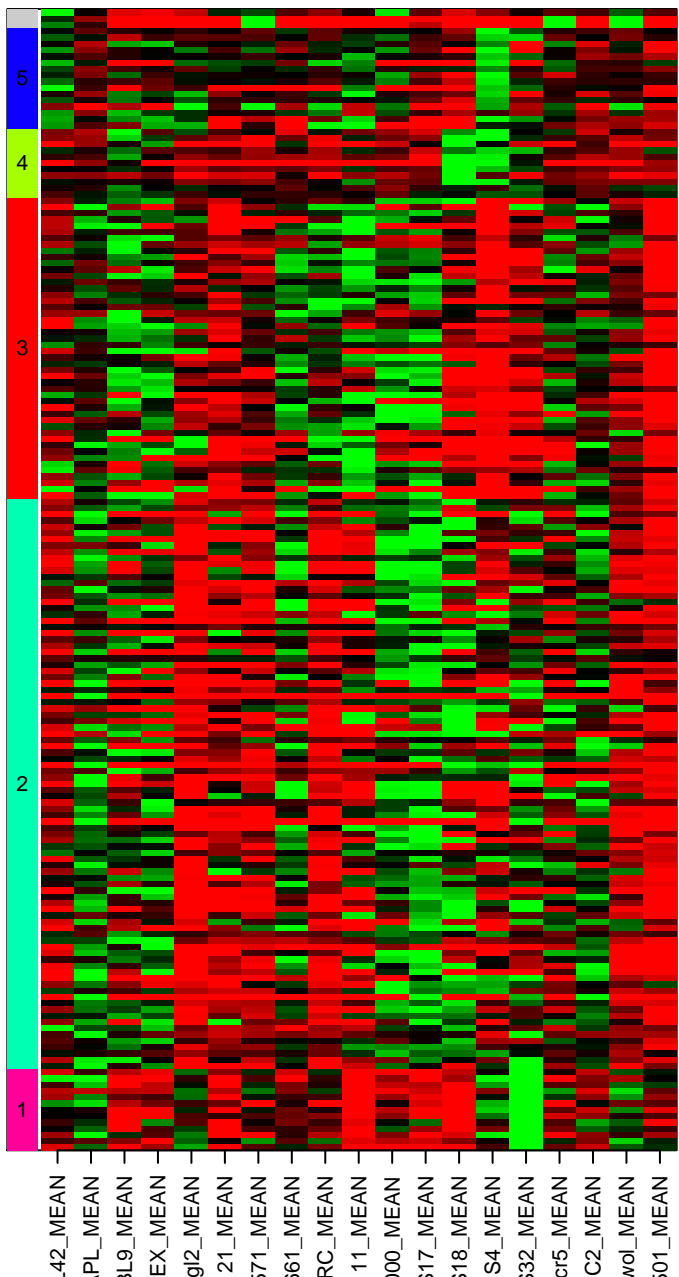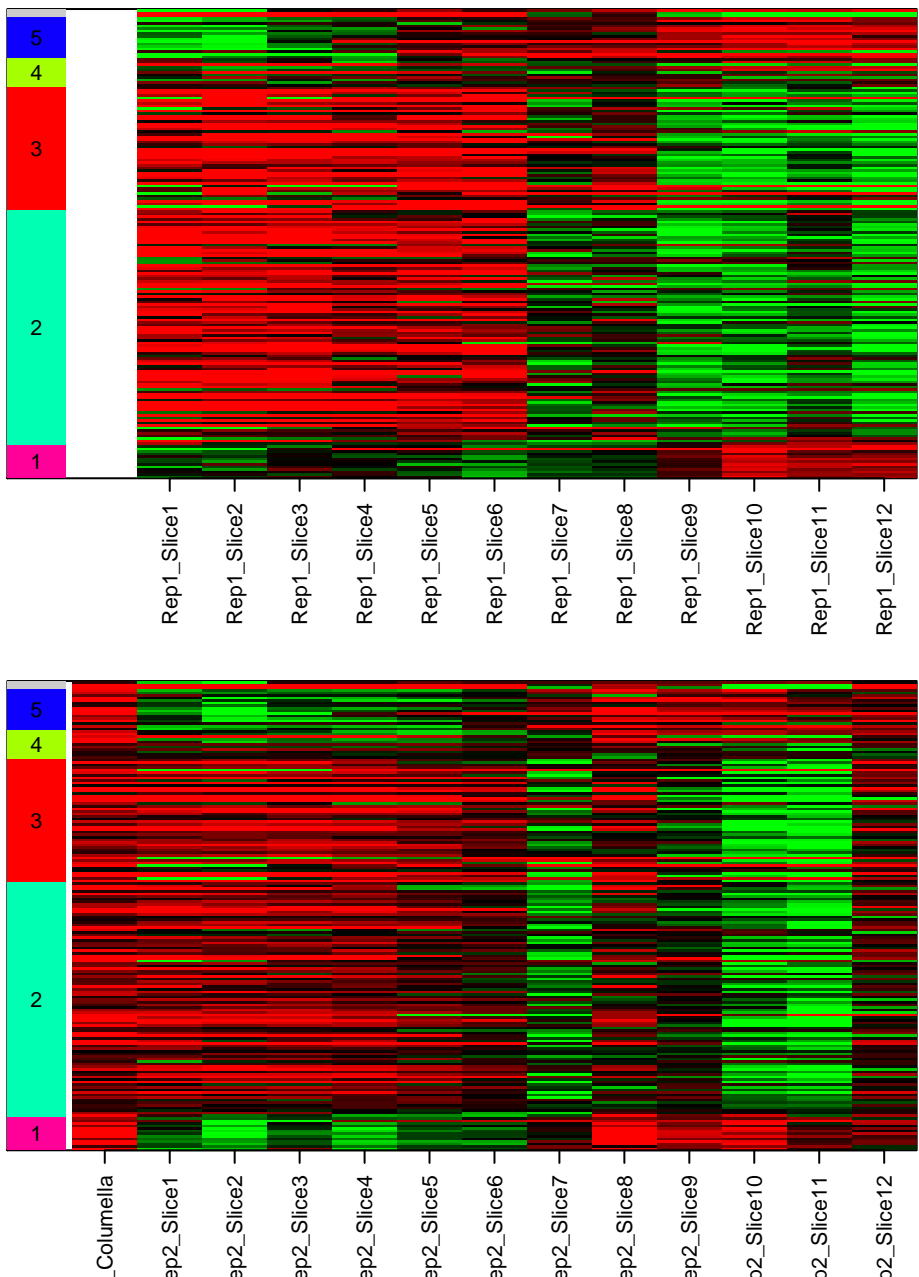

Shift -2

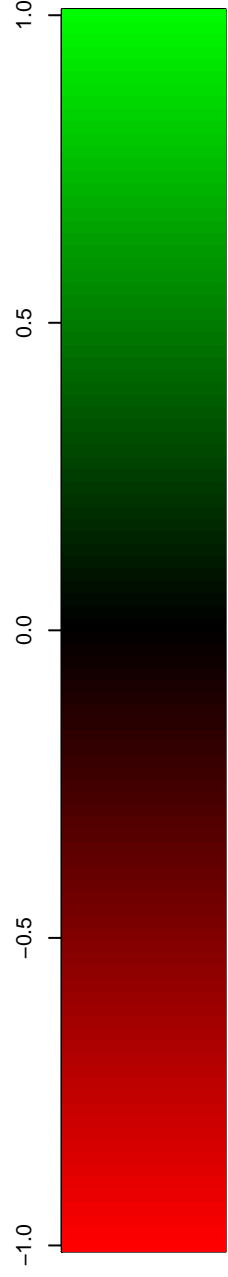

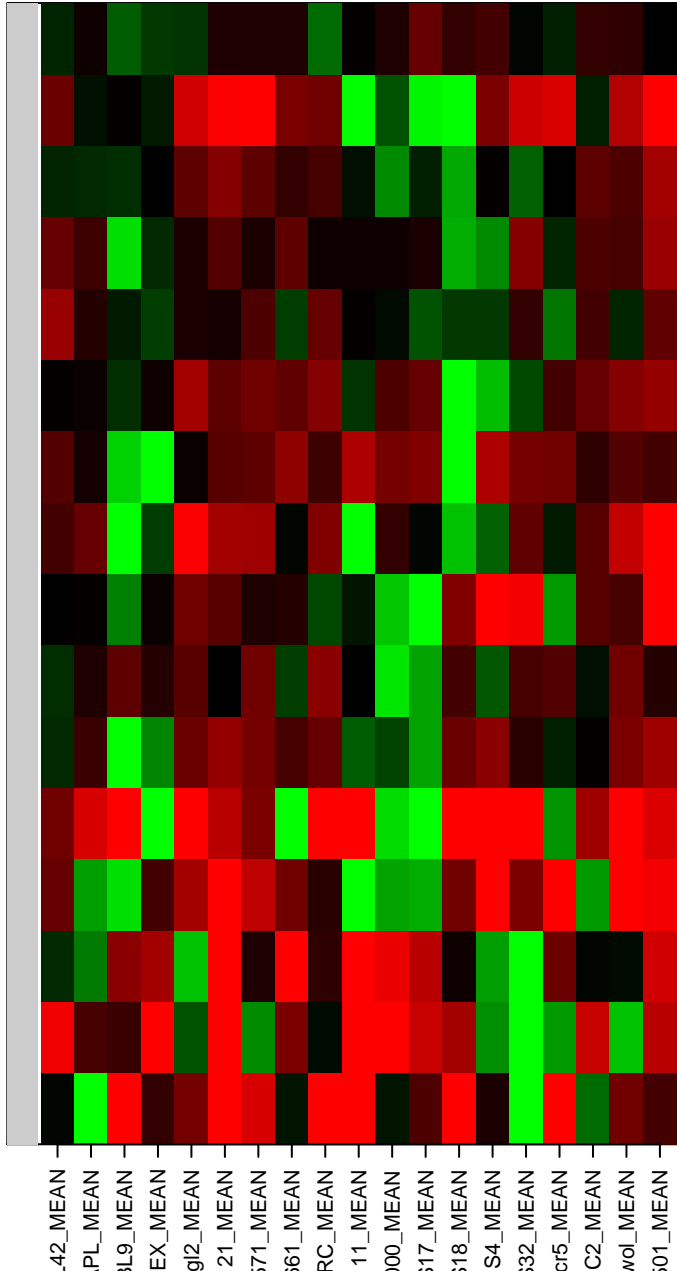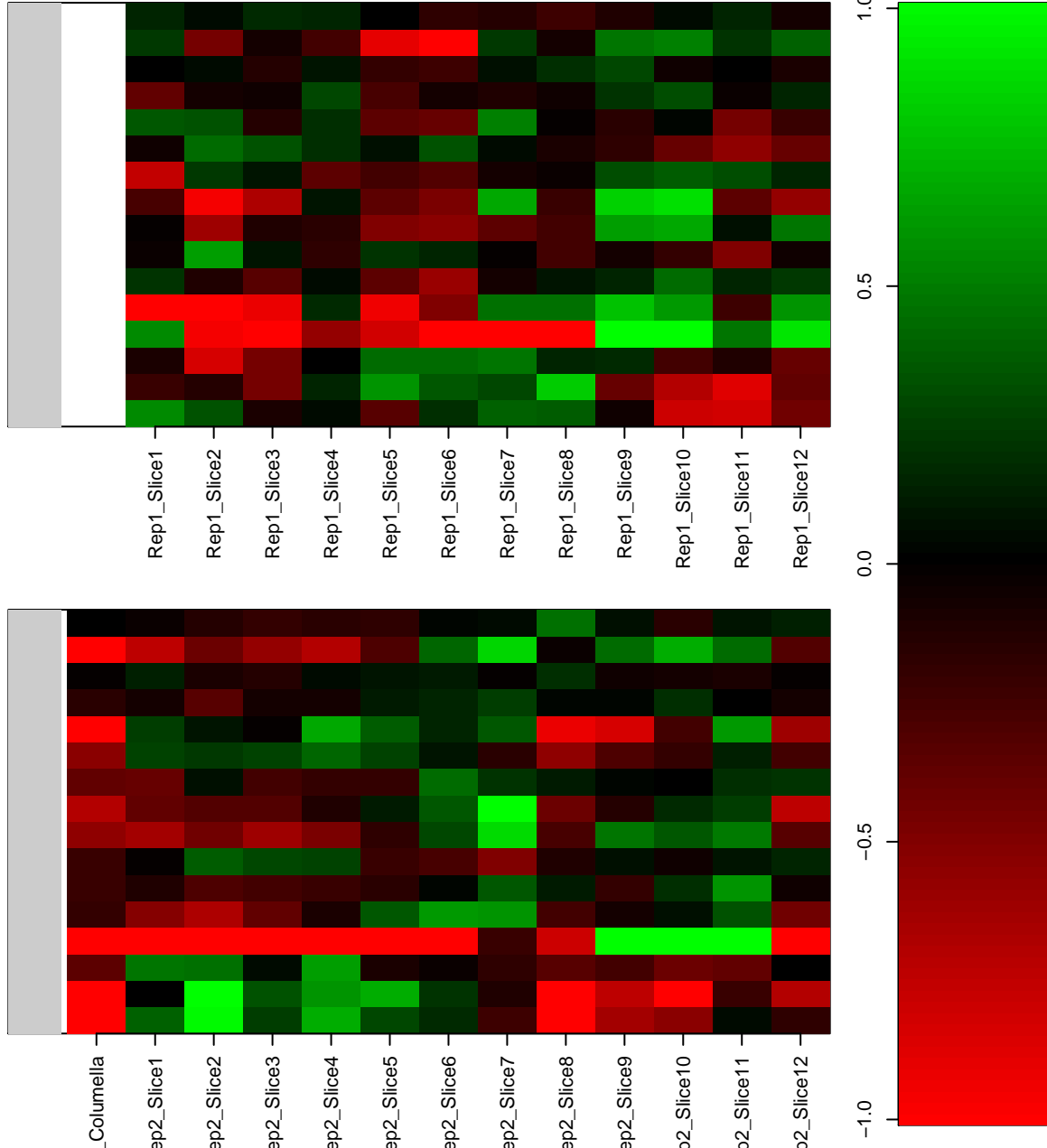

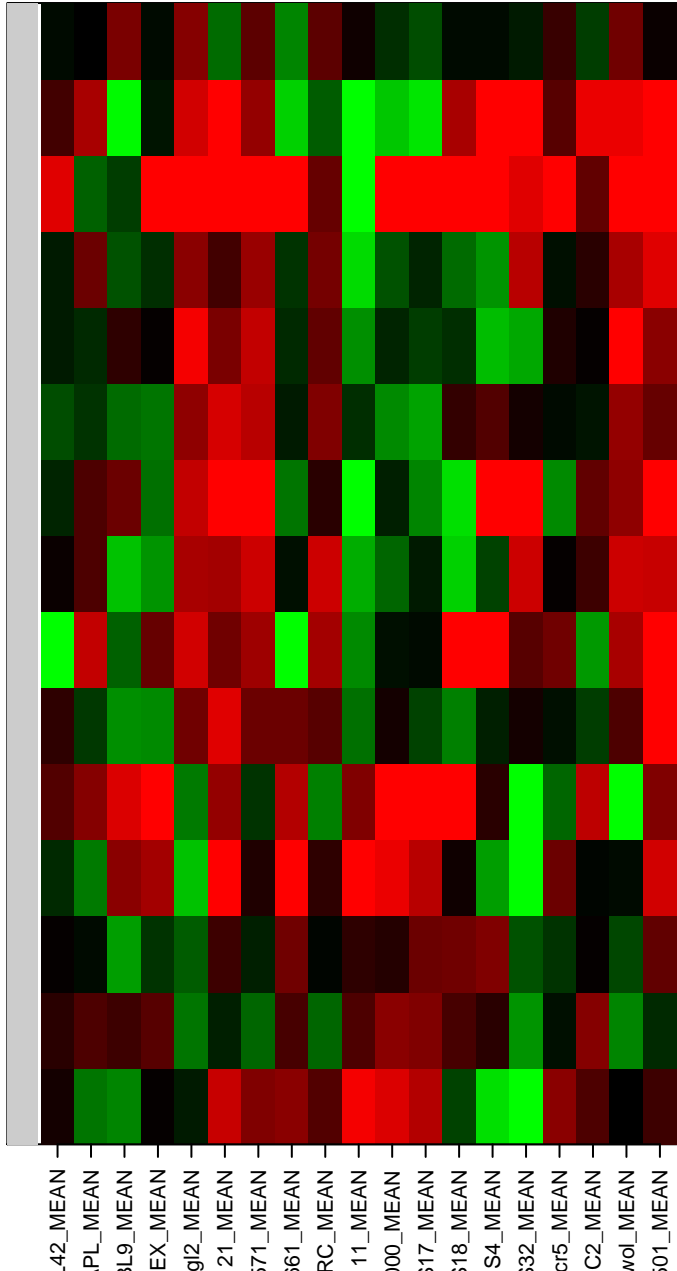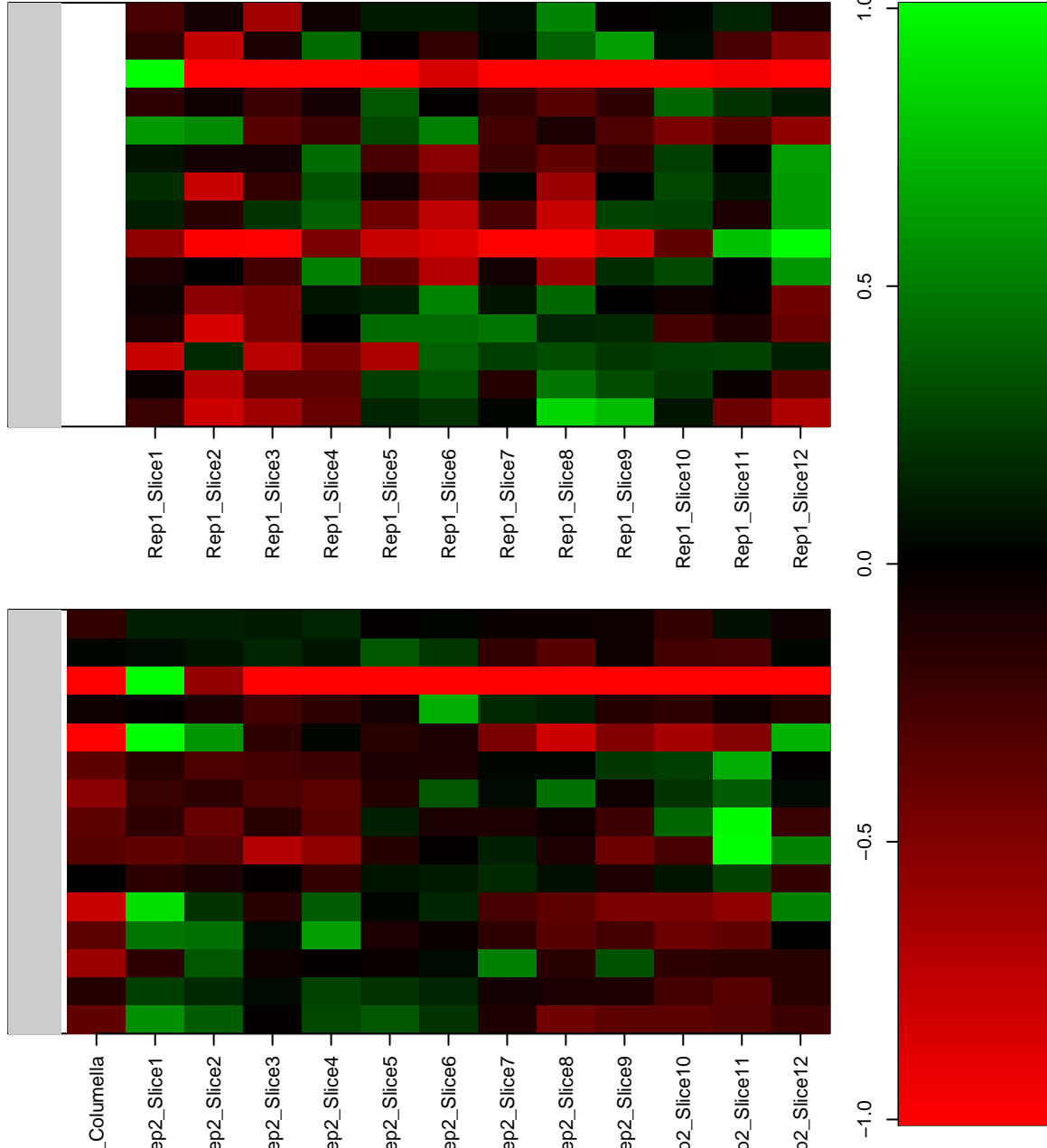

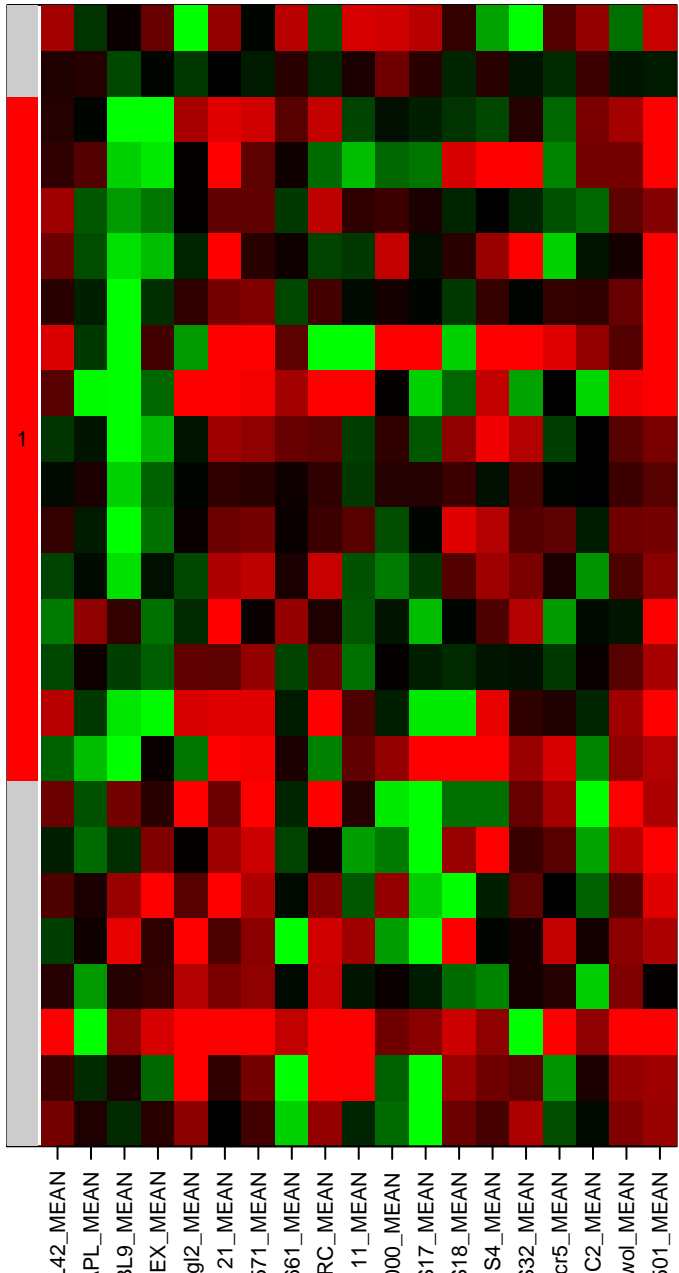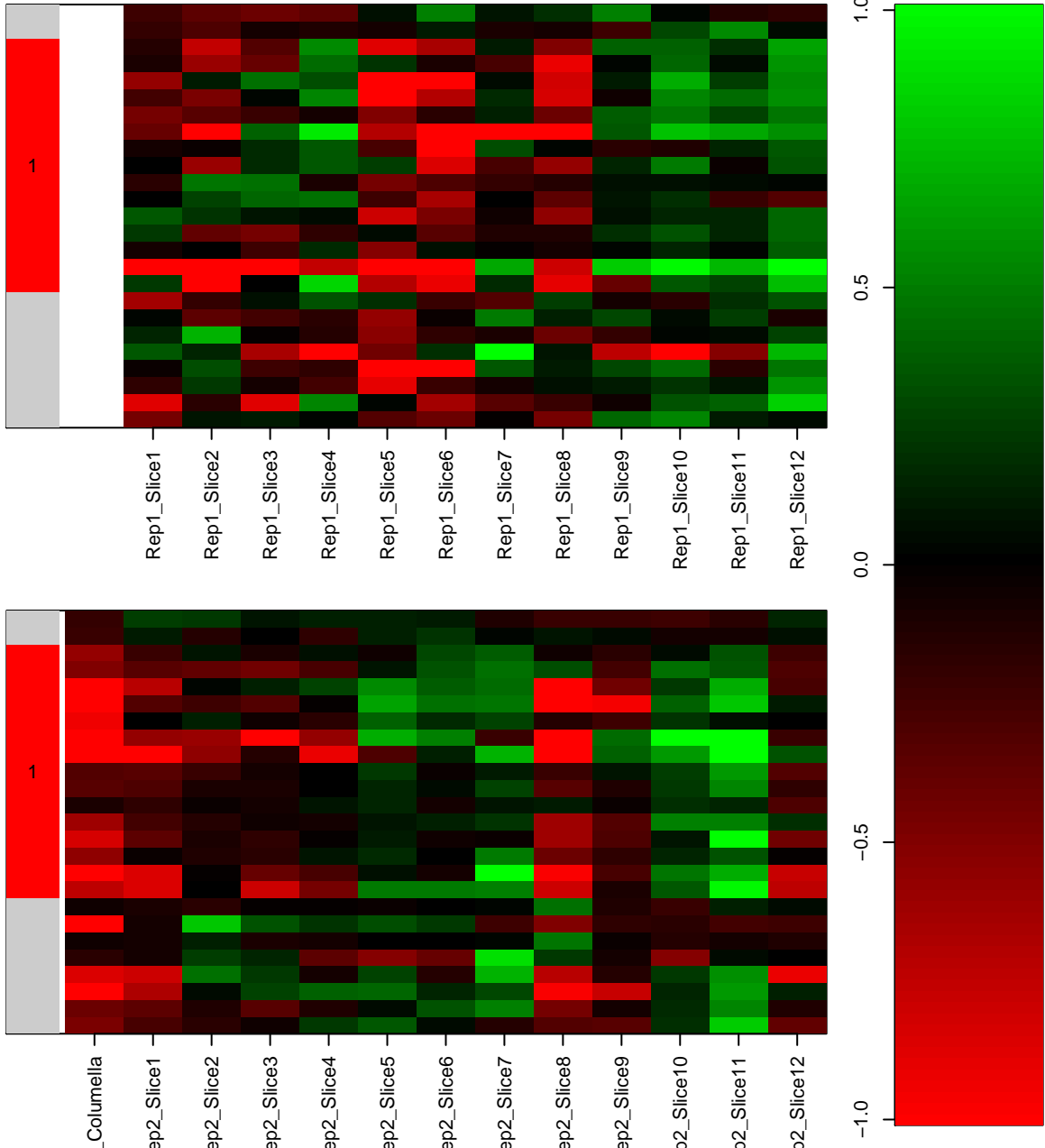

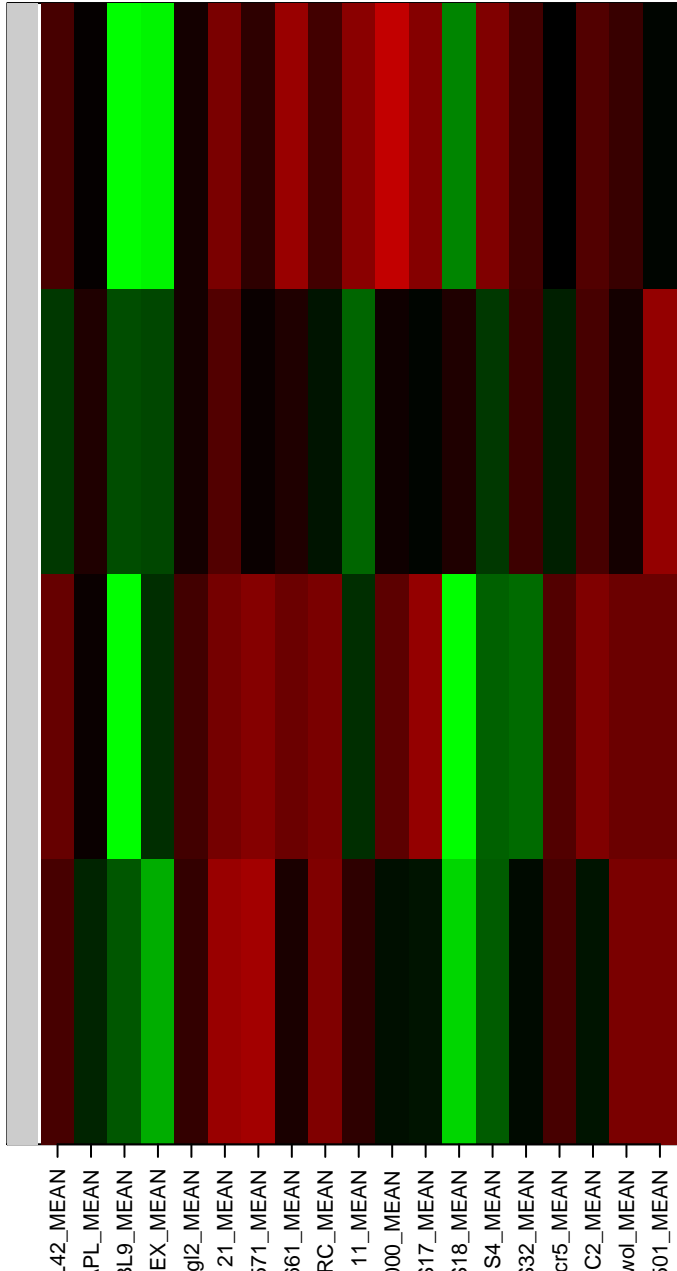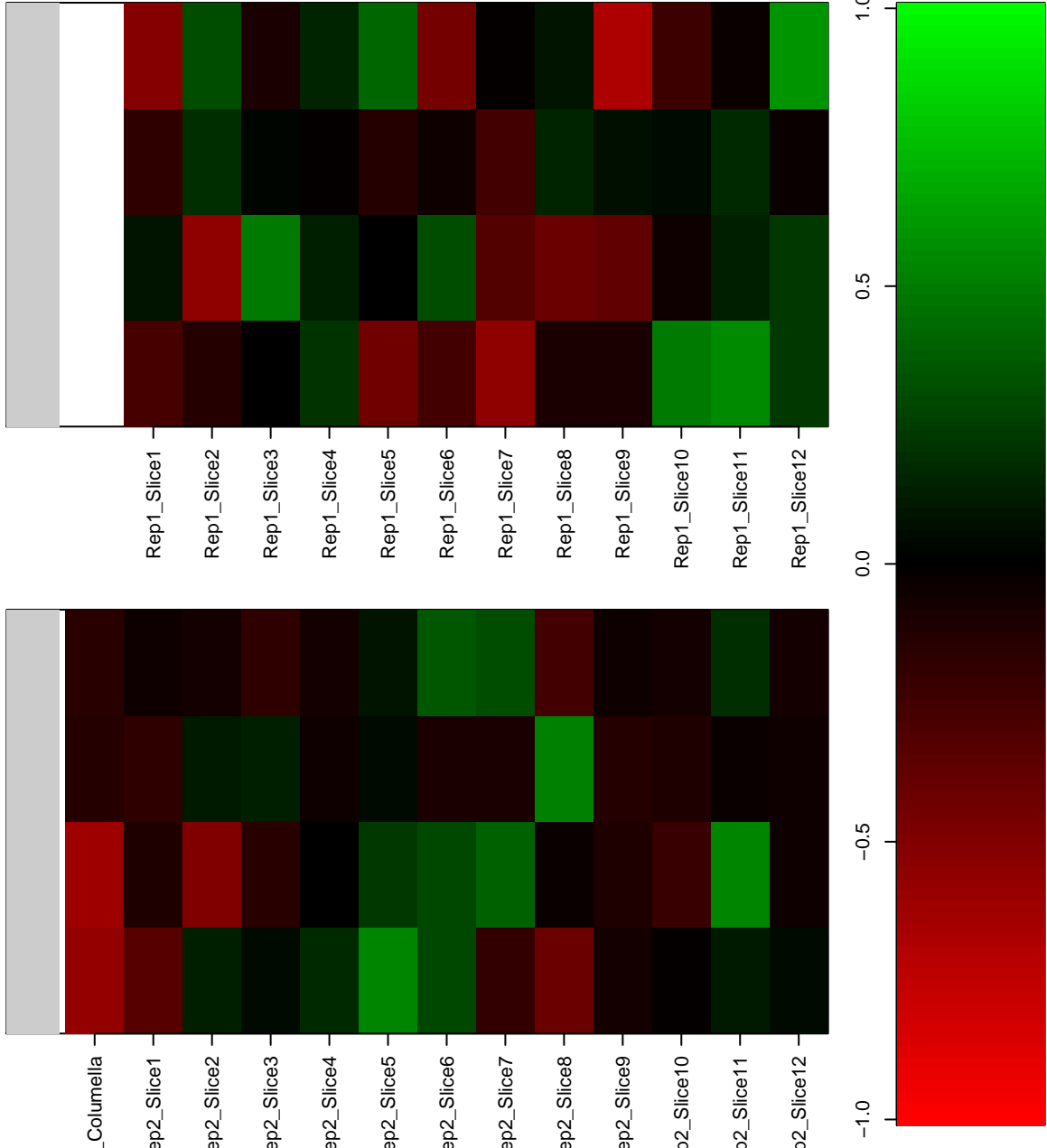

Supplement: Additional File 1 — Clustering of shift profiles identifies spatiotemporally regulated modules of genes. A figure showing clustering of shift profiles identifies spatiotemporally regulated modules of genes for shifts of +2, +3, +4, -2 and -5. For all shifts, relative expression by marker line is visualized in the left heatmap, and relative expression by longitudinal section in the two roots is visualized in the right heatmaps. The relative expression scale is visualized on the right. If clusters with greater than ten members were identified, these are indicated on the left side of each heatmap. [file 1471-2164-11-381-S1.PDF]
